# Evolution of Char Structure and Its Influence on Reactivity During Biomass Pyrolysis: Spatial Scale Effects from Pellet Size to Intra-Pellet Location

**DOI:** 10.3390/polym18080964

**Published:** 2026-04-15

**Authors:** Huping Liu, Yun Yu, Jingyi Wu, Jingchun Huang, Wei Hu, Li Xia, Yu Ru, Maolong Zhang, Minghou Xu, Yu Qiao

**Affiliations:** 1 State Key Laboratory of Coal Combustion, Huazhong University of Science and Technology, Wuhan 430074, China; 2 Discipline of Chemical Engineering, Western Australian School of Mines: Minerals, Energy and Chemical Engineering, Curtin University, GPO Box U1987, Perth, WA 6845, Australia; 3 School of Energy and Power Engineering, Huazhong University of Science and Technology, Wuhan 430074, China; 4 Hubei Yuneng Low Carbon Technology Co., Ltd., Wuhan 430070, China; 5 Beijing Huaneng Yangtze Environmental Technology Research Institute Co., Ltd., Beijing 102209, China

**Keywords:** biomass, pyrolysis, intra-pellet, char structure, intrinsic reactivity

## Abstract

Biomass, composed of natural polymers such as cellulose, hemicellulose, and lignin, can be converted into circular chemical feedstocks through thermochemical conversion processes like pyrolysis. Char conversion is the rate-limiting step in the thermochemical conversion process, and thus, char reactivity is essential for determining the overall efficiency of pellet-based thermochemical processes. Pyrolysis experiments were conducted on rice straw pellets of different sizes (i.e., 8, 10, and 12 mm) in a vertical quartz tube reactor at 700 °C, and then the chemical structure of chars sampled at different stages and locations within a 10 mm pellet was analyzed using Raman spectroscopy and Fourier transform infrared spectroscopy (FTIR). The results indicate that increasing the pellet size facilitates the growth of polycyclic aromatic structures, as evidenced by the observed variations in the abundance of typical aromatic compounds in bio-oil. This also promotes volatile–char interactions, leading to greater deposition of large aromatic structures on the char surface, thereby enhancing char aromatization. Analogous to the spatial scale effect of pellet size on char structure, the evolution of the char structure within a single pellet exhibits distinct spatial heterogeneity during the initial devolatilization and subsequent char aromatization stages due to the location-dependent coupling of heat/mass transfer limitations and aromatization reactions during pyrolysis. Furthermore, the spatiotemporal evolution of the char structure leads to differences in the specific reactivity: during the devolatilization stage at 75 s, the center exhibits the highest reactivity, whereas the outer surface becomes the most reactive in the subsequent char aromatization stage at 300 s.

## 1. Introduction

The excessive consumption of fossil fuels has caused severe environmental degradation and global warming in recent years. In response, carbon-neutral biomass energy, with its abundant reserves, is particularly favored as a promising sustainable alternative to fossil fuels [1]. However, the inherent disadvantages of its loose structure and low energy density have restricted energy utilization for large-scale industrial applications. To address these limitations, bulk biomass can be densified into cylindrical pellets through mechanical compression [2,3]. Pyrolysis not only produces char but also serves as the initial stage in biomass combustion and gasification processes [4]. Compared with pulverized biomass particles, more significant temperature gradients exist within the compacted pellet, thereby leading to greater heterogeneity in the char structure and reactivity [5,6]. Char conversion is widely recognized as the rate-limiting step in thermal conversion processes, such as combustion and gasification, making its reactivity a critical parameter for enhancing overall process efficiency and product quality [7].

It is well established that char reactivity is governed by multiple factors, among which the char structure, inorganic species (mainly alkali and alkaline earth metals), and reaction atmosphere are particularly well documented [8,9,10,11,12,13]. Alkali and alkaline earth metals, which are inherently present in fuel feedstocks, can act as intrinsic catalysts in volatile–char interactions to enhance reactivity [8,9,10]. Chars prepared in CO_2_ exhibit higher reactivity than those prepared in N_2_, with little influence attributed to the combustion atmosphere [11,12]. Moreover, CO_2_ and H_2_O may compete for active sites on the char surface, potentially influencing char reactivity [13].

Besides inorganic species and the reaction atmosphere, the chemical structure of char plays a critical role in determining char reactivity during biomass thermal conversion [14,15,16,17]. In response, the chemical structure characteristics of char, including carbon skeleton structures (e.g., aromatic rings) and oxygen-containing functional groups, are analyzed using Raman and FTIR spectroscopy [18,19]. Raman parameters can reflect how char reactivity depends on volatile–char interactions or the condensation reactions of smaller aromatic rings into larger aromatic structures [20]. It is found that small aromatic rings (3–5 fused rings) are an important factor in determining char reactivity [20], and volatile–char interactions also facilitate aromatic growth through ring condensation, thereby influencing char structural evolution and char reactivity [21,22,23,24]. Furthermore, the variations in oxygen-containing functional groups, resulting from volatile–char interactions, also provide an insight into the evolution of char chemical structures [25,26]. Structural evolution toward small aromatic rings is evidenced by the increase in aromatic C=C bonds at the expense of olefinic C=C, driven by cyclization and aromatization reactions [27]. The dynamic evolution of functional groups mirrors the rearrangement of the carbon skeleton at the molecular scale and further influences the subsequent reactivity of the char [10,28].

However, previous studies on char structural evolution have primarily focused on the overall structure of bulk biomass particles or single pellets, overlooking the spatiotemporal evolution of the char structure and its role in governing reactivity within a pellet. During single-pellet pyrolysis, pronounced intraparticle heat and mass transfer limitations prolong the residence time of primary volatiles, promoting their secondary reactions [29,30]. The increased secondary reactions within the pellet alter the secondary char formation pathways, ultimately leading to a more heterogeneous and complex char structure. Chen et al. [31] proposed an evolution route for the char chemical structure during pellet pyrolysis using only the area ratios of Raman characteristic peaks. It was found that the temporal and spatial evolution of the char chemical structure within the pellet was governed by heat and mass transfer limitations, specifically in secondary reactions such as volatile–char interactions or the progressive condensation and growth of aromatic rings [32]. As a result, the specific variations in product distribution have not been fully elucidated, leaving a gap in understanding how these reactions influence char structural evolution. Just as heat and mass transfer resistances vary spatially within a single pellet, differences in pellet size impose varying degrees of transport limitations [33]. This scale effect influences pyrolysis product distribution and char structural evolution, ultimately dictating char reactivity. Thus, a comparative analysis of the influence of pellet size on product distribution and properties is essential for elucidating the underlying spatial scale effect within a single pellet.

This work aims to mechanistically investigate the effect of pellet size (a macro-scale variable) on product distribution and properties, thereby revealing how spatial scale influences the heterogeneity of the char structure and reactivity within the pellet. Pyrolysis experiments were conducted on single pellets of different sizes in a vertical quartz tube reactor at 700 °C. All products, including bio-oil, light gases, and solid char, were collected and characterized. To gain deeper insight into the differences in char structural evolution and reactivity, pyrolysis chars sampled at different stages and locations within a representative 10 mm pellet were analyzed using Raman spectroscopy and FTIR spectroscopy. This study will provide critical insights into the spatial scale effect on the heterogeneity of the char structure and reactivity within pellets, which is vital for optimizing the pyrolysis process and enhancing the overall conversion efficiency of pellet-based thermochemical processes.

## 2. Experimental

### 2.1. Sample Preparation and Properties

Rice straw from a farm in Hubei was used in this study. Table 1 summarizes the proximate and ultimate analysis results of the raw sample. The proximate and ultimate analyses were performed in accordance with the standards GB/T 28731-2012 [34] and ASTM D5373 [35], respectively. Firstly, the raw rice straw was ground and sieved to a particle size between 75 and 180 µm using a grinder and then dried in an oven at 105 °C for 24 h. In addition, deionized water was added to produce a moisture content of 12 wt.% for subsequent densification treatment. Then, 1.85 ± 0.05 g of the rice straw sample was compacted using a universal material testing machine (WDW-50E, Jinan Shijin Group Co., Ltd., Jinan, China) under a pressure of 125 MPa to prepare a cylindrical pellet. These pellets exhibited diameters of 8, 10, and 12 mm, respectively, with a uniform height of 20 ± 0.1 mm. The density of all the pellets used in the experiments was 1.17 ± 0.01 g/cm^3^, calculated from the measured mass and volume of each pellet [36].

### 2.2. Pyrolysis Experiments

**Figure 1:**
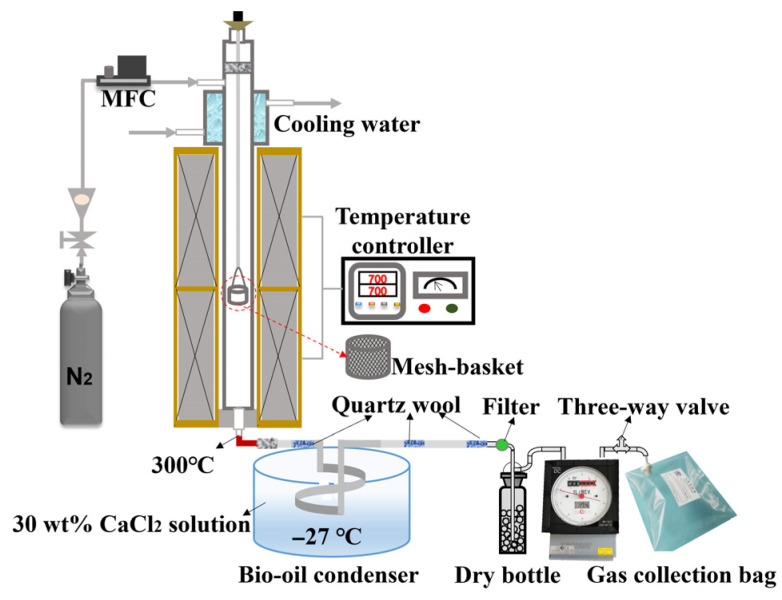
Schematic diagram of the pyrolysis system.

The single-pellet pyrolysis experiments were conducted using a vertical fixed-bed system (see Figure 1) at 700 °C under a N_2_ (99.999%) atmosphere. The vertical fixed-bed system was mainly composed of a temperature control unit, a gas supply unit equipped with a mass flow controller, a quartz reactor unit with water cooling, a bio-oil collection unit, and a gas collection unit.

**Figure 2:**
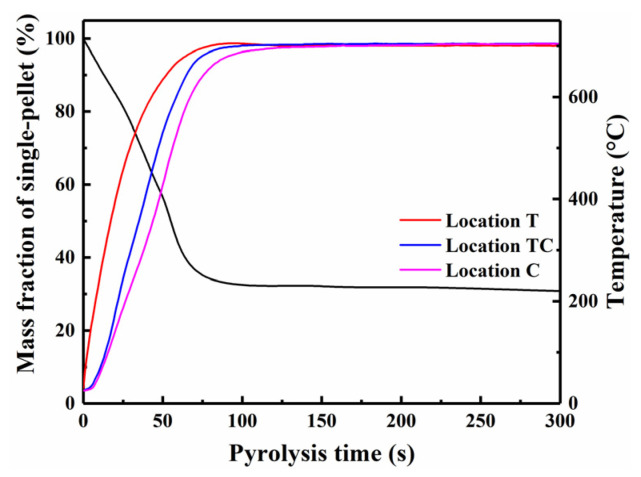
Mass loss of single-pellet and temperature profiles at different locations.

Before the experiments, the furnace was heated to 700 °C, and pre-heated N_2_ was continuously supplied through the top side of the quartz tube reactor at a flow rate of 3 L/min to maintain an inert atmosphere. A mesh basket (woven from 0.1 mm wire) loaded with a single pellet was placed into the quartz tube (maintaining a constant temperature zone) and connected to a Pt wire fixed by a high-temperature silicone cork at the top inlet of the reactor. Pellets of different diameters (i.e., 8, 10, and 12 mm) were maintained in a constant temperature zone for 5 min. During the experiments, the bottom outlet of the reactor was connected in series to a spiral condenser in a CaCl_2_ solution bath (approximately −27 °C) for the collection of condensed liquid products, and the interface at the outlet of the reactor was wrapped with electric heating tape to maintain a constant temperature of 300 °C. The non-condensable gas was collected using a Teflon gasbag(Dalian Haidi Technology Co., Ltd., Dalian, China), and its total volume was determined using a dry gas cumulative flowmeter (Shinagawa DC-1, Shinagawa Corporation, Tokyo, Japan). After 5 min of pyrolysis, the mesh basket was quickly moved into a water-cooling jacket (approximately 60 °C) for quenching [37], followed by disconnection of the reactor–condenser interface. The charred pellet was then purged with N_2_ (8 L/min) to an ambient temperature. Furthermore, for pellets with a 10 mm diameter, temperature profiles at locations T, TC, and C within the pellet were measured through separate experiments, as described in detail in our previous study [38]. Reaction times of 28, 75, 110, 200, and 300 s were selected as quenching points (see Figure 2), and then the pellet char was rapidly quenched with N_2_ upon reaching each target pyrolysis time. Finally, the pellet char was carefully collected to preserve its original morphology and stored hermetically for further analysis. At the end of each experiment, the yields of solid char and liquid products were calculated by the weight differences between the single pellets and the corresponding collection container (i.e., spiral tube and dry filter bottle) before and after each experiment, respectively [39].

### 2.3. Sample Analytical Methods

#### 2.3.1. Sampling Process for Char Samples at Specific Locations

**Figure 3:**
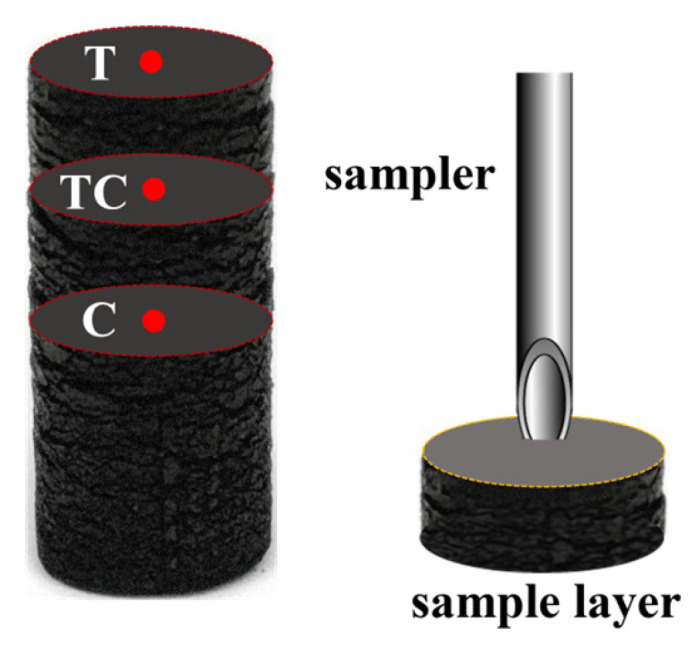
Process of sampling at specific locations within single-pellet char (10 mm in diameter).

The char samples at specific locations within the single-pellet char (10 mm in diameter), produced at different times (i.e., 28, 75, 110, 200, and 300 s) during pyrolysis at 700 °C, were obtained using a sampler. As shown in Figure 3, these locations specifically included T (center at the top surface), TC (center at the 1/4 cross section from the top), and C (center at the middle cross section). When sampling at each location, the single sampling quantity was approximately 5 mg to ensure uniformity of all the collected samples at each location. Finally, the collected char samples and pellet char (i.e., 8, 10, and 12 mm) were ground to <50 µm using a mortar for subsequent analysis.

#### 2.3.2. Characterization of Solid Char Products

The raw rice straw and its char samples at different stages and locations within the pellet were dissolved by the acid digestion method, and then the solutions were analyzed via inductively coupled plasma optical emission spectroscopy (Prodigy Plus, Teledyne Leeman Labs, Hudson, NH, USA) to determine the contents of alkali and alkaline earth metals [40].

The carbon structure of the solid char was analyzed using a Raman spectrometer (LabRAM HR800, Horiba Scientific, Palaiseau, France) with an excitation wavelength of 532 nm. The evolution of the carbon structure was characterized by specific Raman bands in the wavenumber range of 800–1800 cm^−1^. The original Raman spectra were curve-fitted into 10 Gaussian bands using PeakFit v4.12 software (Systat Software, San Jose, CA, USA), following the detailed fitting method from the previous literature [22,23,41,42]. A high-quality curve fitting with an R^2^ value exceeding 0.95 confirmed the reproducibility of the derived spectral parameters. For these 10 bands, the D band (1300 cm^−1^) represented the carbon structure with no less than six aromatic fused rings, and the G band (1590 cm^−1^) mainly denoted aromatic ring quadrant breathing due to the relatively low intensity of the graphite structure in these chars [42]. Meanwhile, the G_r_ (1540 cm^−1^), V_l_ (1465 cm^−1^), and V_r_ (1380 cm^−1^) bands together reflected the carbon structure of smaller aromatic ring systems with 3–5 fused rings. Therefore, the area ratio of the D band to the G band (A_D_/A_G_) identified the complexity of the carbon structure in chars, with a larger ratio indicating a more significant aromatization effect. In addition, the area ratio (A_D_/A_(Gr+Vl+Vr)_) of the D band to the combined (G_r_ + V_l_ + V_r_) band indicated the relative ratio between the large (≥6 fused rings) and the small (3–5 fused rings) aromatic ring systems in the chars.

The functional groups of solid char were analyzed using Fourier transform infrared spectroscopy (Nicolet iS50R, Thermo Fisher Scientific, Waltham, MA, USA) in the wavenumber range of 600–4000 cm^−1^ with a resolution of 4 cm^−1^. Briefly, a small amount of the sample (approximately 1.3 mg) was mixed with pure KBr (approximately 130 mg) by grinding it in an agate mortar, followed by testing [28]. All spectra were corrected for background and baseline and then normalized to the unit mass of the char sample for semi-quantitative analysis.

The specific reactivity of the char was determined using a thermogravimetric analyzer (STA 409, NETZSCH-Gerätebau GmbH, Selb, Germany) under an air atmosphere. To minimize the effect of oxygen chemisorption on reactivity measurements, a gas mixture of 5% O_2_ in N_2_ was used at a constant flow rate of 30 mL/min under 400 °C for char particles (<75 μm), similar to a differential reactor [9,10]. Briefly, about 5 mg of char was first heated to 120 °C under a pure N_2_ flow and held for 30 min. Then, the char was heated to 400 °C at a rate of 20 °C/min, at which point the gas was switched from N_2_ to 5% O_2_ in N_2_, and the char was held isothermally at 400 °C for 4 h. The specific reactivity (R, min^−1^) of a char at any time was calculated from the differential mass loss data (dW/dt) using the following formula: R = −(1/W) × (dW/dt), where W is the mass (dry and ash-free) of the char at any time t (min).

#### 2.3.3. Characterization of Liquid Products

The liquid products in the spiral tube were mainly composed of organic phase products and condensed water. The water content in the liquid product was determined using a Karl Fischer moisture titrator (899 coulometer, Metrohm AG, Herisau, Switzerland). The bio-oil yield was calculated by the difference between the total liquid yield and the water content [39]. The bio-oil components were analyzed by gas chromatography–mass spectrometry (Agilent 7890A/5975C, Agilent Technologies, Santa Clara, CA, USA) with a HP-5 MS column (30 m × 0.2 mm × 0.25 μm). For each analysis, the bio-oil was first diluted with acetone, and 1 μL of the diluted solution was injected into the port without being split. The temperature program was as follows: it was first kept at 40 °C for 5 min, then increased by 5 °C/min to 280 °C and held for 20 min. The compounds in the bio-oil were identified using the National Institute of Standards and Technology (NIST) library. The major compounds were semi-quantified based on the peak area, normalized to per gram of the dry feedstock. The relative peak area became a semi-quantitative method to estimate the “yield” of compounds in the bio-oil products [39].

#### 2.3.4. Characterization of Gas Products

The gas products (i.e., CO, H_2_, CH_4_, CO_2_, C_2_H_4_, C_2_H_2_, and C_2_H_6_) were quantitatively analyzed using gas chromatography (Agilent 3000A, Agilent Technologies, Santa Clara, CA, USA). The yield of each gas was calculated by multiplying the total gas volume by the respective density of each ideal gas component.

## 3. Results and Discussion

### 3.1. Effect of Pellet Size on Product Distribution and Characterization During Pyrolysis

#### 3.1.1. Yield Distribution of Pyrolysis Products

Table 2 presents the mass yields of bio-oil, moisture, gas, and solid char during different-sized (i.e., 8, 10, and 12 mm) single-pellet pyrolysis at 700 °C. Typically, volatiles were primarily produced from the pyrolysis of cellulose and hemicellulose, whereas solid char and light gases were mainly derived from the pyrolysis products of lignin [43]. In this study, the mass balance, calculated as the sum of bio-oil, moisture, gas, and solid char normalized to the feedstock mass, ranged from 95.68% to 104.86%. With an increase in pellet size from 8 to 12 mm, the yields of solid char and gas increased by 1.16% and 0.67%, respectively, whereas the bio-oil yield decreased by 2.66%. Theoretically, pellet size significantly influenced heat and mass transfer during pyrolysis, resulting in a gradual decrease in the heating rate from the outer surface to the inner layer. Larger pellets exhibited greater temperature gradients and longer residence times for the volatiles, both of which promoted secondary reactions [33,44]. Therefore, increasing single-pellet size favored the formation of a secondary char and light gas by promoting bio-oil consumption.

#### 3.1.2. GC/MS Analysis of the Bio-Oil

**Figure 4:**
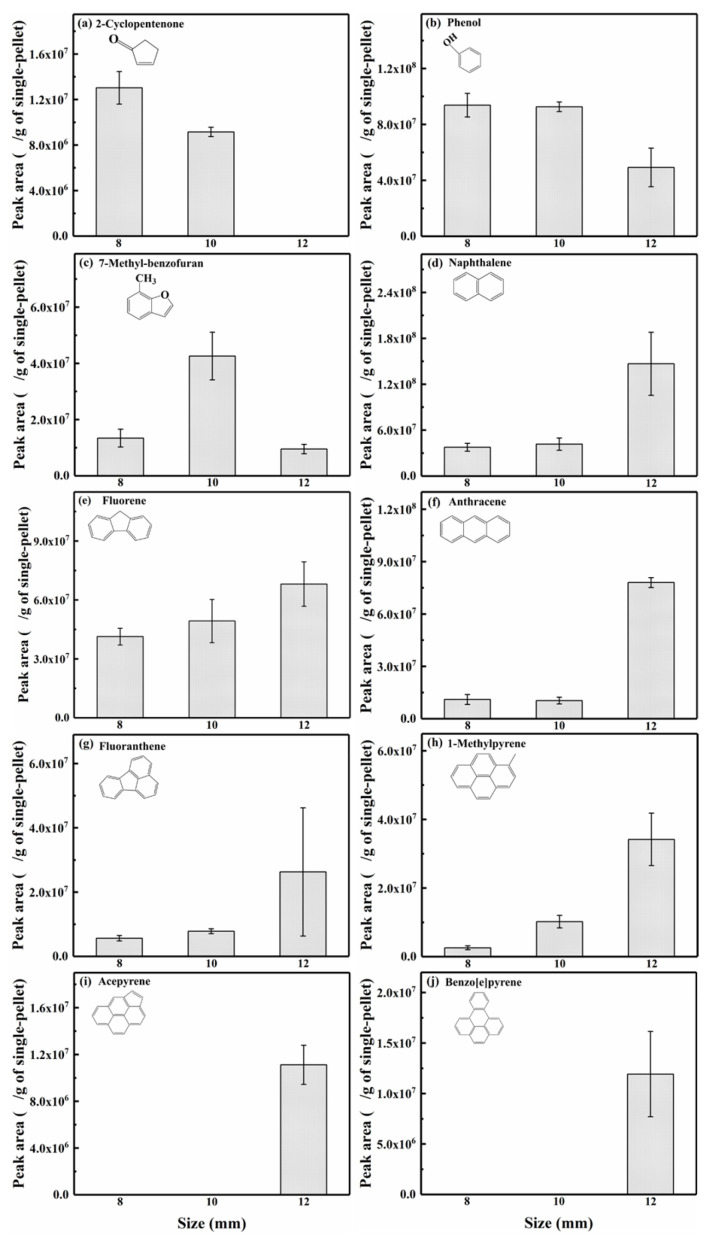
Relative peak areas of some typical aromatic products in bio-oil.

The light components in the bio-oil produced from different-sized (i.e., 8, 10, and 12 mm) single-pellet pyrolysis at 700 °C were analyzed by GC-MS. In this study, it was found that almost all light components in the bio-oil possessed ring structures, particularly aromatic rings. Figure 4 shows the relative peak areas of some typical aromatic compounds in the bio-oil, indicating that larger pellets were more conducive to the condensation or growth of fused aromatic rings. Typical 1-ring compounds (e.g., 2-cyclopentenone and phenol) generally exhibited a decrease in peak area with increasing pellet size (8–12 mm). For two typical 2-ring compounds, 7-methylbenzofuran exhibited an initial increase followed by a decrease in peak area with size, whereas naphthalene abundance increased with size. This reduction in peak area for these compounds was attributed to decarbonylation, demethylation, and dehydrogenation reactions [45,46]. Compared to 1- and 2-ring compounds, 3- and 4-ring compounds were not only products but also acted as reactants in subsequent condensation reactions [47]. As pellet size increased, fluorene, anthracene, fluoranthene, and 1-methylpyrene exhibited a comparable trend in peak area to that of naphthalene. For 5-ring compounds, acepyrene and benzo[e]pyrene were observed only in bio-oil produced from the pyrolysis of a 12 mm pellet at 700 °C. The variations in peak areas of the above typical compounds in bio-oil indicated that larger pellets prolonged the residence time of volatiles and promoted the condensation or polymerization reactions of small fused aromatic rings to form larger aromatic ring structures.

#### 3.1.3. Analysis of Gas Products

**Figure 5:**
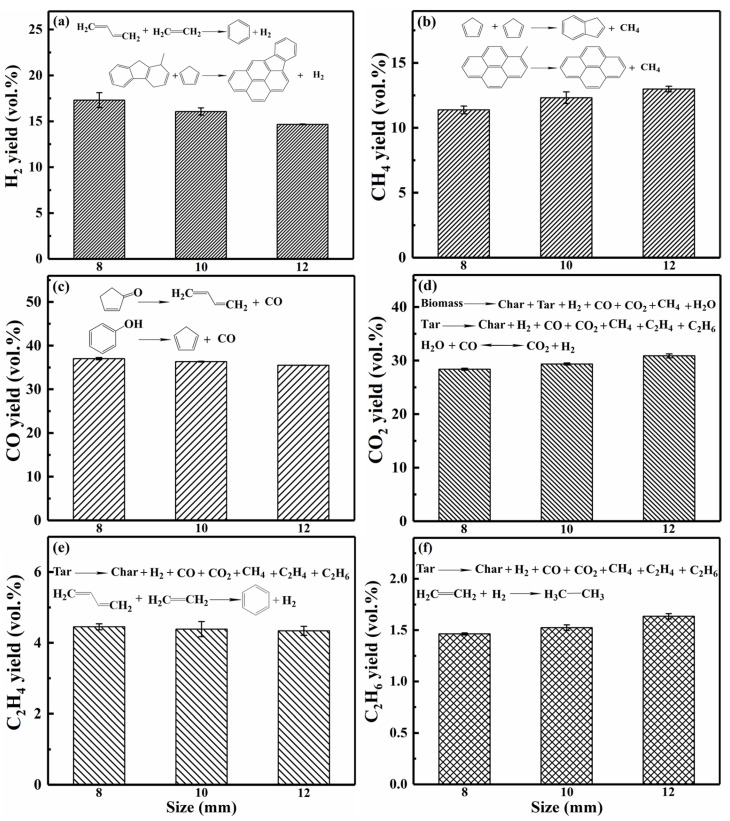
Gas yields produced from single pellets of three different sizes.

Figure 5 illustrates the composition and volume yield of the main gas products (i.e., H_2_, CH_4_, CO, CO_2_, C_2_H_4_, and C_2_H_6_) generated by the three pellet sizes. It was observed that the pellet sizes, ranging from 8 to 12 mm, had no significant effect on gas composition and yield. Larger pellets slightly promoted the release of CH_4_, CO_2_, and C_2_H_6_ but decreased that of H_2_, CO, and C_2_H_4_. Specifically, the H_2_ yield decreased from 17.30% (8 mm) to 14.66% (12 mm). Hydrogen radicals acted as pivotal intermediates in the stepwise cyclization of aromatic precursors, thereby consuming a portion of H_2_ [47,48]. Similarly, CO and C_2_H_4_ yields showed minor reductions of approximately 1.51% and 0.12%, respectively, with increasing size. In contrast, CH_4_, CO_2_, and C_2_H_6_ yields increased by 1.60%, 2.49%, and 0.17%, respectively. The results indicated that larger pellets were conducive to the formation of stable saturated light gases [49]. Most light gases were produced from the pyrolysis products of lignin in the biomass. Nevertheless, some secondary reactions, such as the polymerization or condensation of small fused rings, can alter the gas reaction pathway and thus influence the yield [47,50]. Larger pellets prolonged the residence time of volatiles within the pellets and enhanced secondary reactions that consumed active free radicals and unsaturated hydrocarbons, resulting in a decrease in CO and C_2_H_4_ yields and an increase in CH_4_, CO_2_, and C_2_H_6_ yields.

#### 3.1.4. Carbon Structure of Solid Char from Raman Spectroscopy

**Figure 6:**
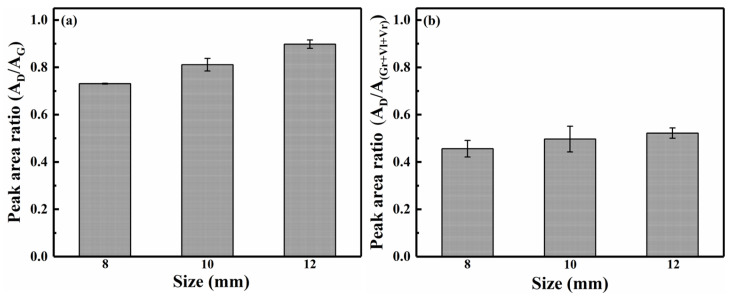
Variations in A_D_/A_G_ and A_D_/A_(Gr+Vl+Vr)_ ratios of char with different pellet sizes.

As shown in Figure 6, the area ratio (A_D_/A_G_) of the carbon structure in the char during pyrolysis at 700 °C increased with increasing pellet size from 8 to 12 mm. This trend suggested an increase in the relative concentration of large aromatic rings with six or more fused benzene rings. Larger pellets led to a slower heating rate and greater mass transfer resistance within the pellet, thereby creating more favorable conditions, such as residence time, for the secondary reactions of volatiles [51,52]. Firstly, the demethylation and dehydrogenation reactions that occurred on branched chain structures facilitated the formation of small aromatic rings (3–5 fused rings). Subsequently, condensation reactions among these small aromatic rings increased the abundance of large aromatic ring structures (≥6 fused rings) at the cost of consuming the small aromatic rings [53]. The volatile–char interactions primarily involved condensation reactions, complemented by radical recombination reactions. During pyrolysis, volatiles underwent substitution and condensation reactions with active sites on the char surface. The condensation reaction served as a core pathway for depositing large aromatic structures, as it can directly integrate small volatile molecules into the aromatic network of the char. Simultaneously, high temperatures generated abundant radicals on both volatiles and char surfaces. These radicals underwent recombination reactions, connecting volatile fragments to the char surface and further promoting large aromatic structure growth. Therefore, as the pellet size increased from 8 to 12 mm, the area ratio (A_D_/A_(Gr+Vl+Vr)_) of the carbon structures slightly increased.

### 3.2. Spatiotemporal Evolution of the Chemical Structure in Char Within a 10 mm-Diameter Pellet

#### 3.2.1. Spatiotemporal Evolution of Carbon Structure from Raman Spectroscopy

**Figure 7:**
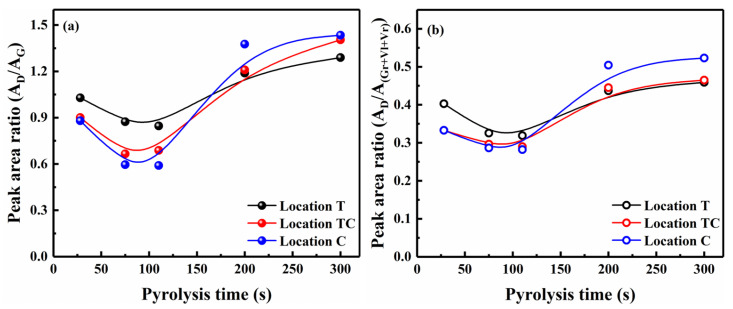
Variations in A_D_/A_G_ and A_D_/A_(Gr+Vl+Vr)_ ratios of char at different locations versus time.

Figure 7 presents the spatiotemporal evolution of the carbon structure in char at locations T, TC, and C versus pyrolysis time—namely, the variations in the A_D_/A_G_ and A_D_/A_(Gr+Vl+Vr)_ ratios. It was observed that the ratios of the char at these three locations decreased initially until 110 s and then increased. The temporal variations indicated a significant transition in the relative concentrations of large and small aromatic ring structures at different locations within the pellet char at 110 s. During the initial stage of pyrolysis (0–110 s), the single pellets underwent rapid heating with a variable heating rate below 700 °C, simultaneously experiencing a significant weight loss before 75 s (see Figure 2). Volatile release primarily occurred within 75 s, driven by the thermal degradation of hemicellulose and cellulose, which generated light gases and small aromatic compounds [49]. Concurrently, the formation of small aromatic rings (3–5 fused rings) through depolymerization and aromatization reactions caused the observed decrease in the A_D_/A_G_ and A_D_/A_(Gr+Vl+Vr)_ ratios depicted in Figure 7 [31]. Subsequently, the continuous increase in temperature from 75 to 110 s, despite limited weight loss, promoted condensation reactions, thereby leading to a more gradual decrease in these ratios. The center temperature lag relative to mass loss completion revealed an asynchrony between heat transfer and reaction kinetics, which reduced the accuracy of the overall reaction rate constant, complicated the application of traditional homogeneous kinetic models, and induced the structural heterogeneity in the char through the enhanced secondary reaction of the volatiles [38]. From 110 to 300 s, the pyrolysis process within the pellet char was primarily governed by mass transfer limitations due to the uniform internal temperature distribution [31]. The small aromatic rings deposited within the pellet char underwent polymerization reactions at the active free radical sites [54]. Thus, the abundance of large aromatic ring structures increased at the expense of small aromatic rings, resulting in increased A_D_/A_G_ and A_D_/A_(Gr+Vl+Vr)_ ratios.

The spatial evolution of the carbon structure within the pellet char at locations T, TC, and C is illustrated in Figure 7. The heat transfer resistance at these locations increased sequentially along the axial direction, from the outer surface toward the center of the inner layer, due to the established axial gradient. It was observed that the A_D_/A_G_ and A_D_/A_(Gr+Vl+Vr)_ ratios of the char at locations T were higher than those at locations TC and C at 75 s. This was attributed to the outer surface location T initially being in contact with high temperatures at the beginning of the heat transfer, with a higher heating rate than at the other two locations (i.e., TC and C) [23,24]. Volatile release primarily occurred within 75 s, concurrent with primary char formation. Higher temperatures favored the formation of large aromatic ring structures, resulting in increased A_D_/A_G_ and A_D_/A_(Gr+Vl+Vr)_ ratios [17,23]. However, the trend in ratios was completely reversed at 300 s compared to 75 s. The evolution of the carbon structure at 75 s was primarily governed by heat transfer resistance, whereas at 300 s, it was predominantly controlled by mass transfer resistance. Smaller mass transfer resistance at the outer surface location T than at TC and C shortened the residence time for volatile condensation and polymerization. The increased abundance of large aromatic ring structures deposited in center location C resulted in higher A_D_/A_G_ and A_D_/A_(Gr+Vl+Vr)_ ratios at 300 s. Therefore, the spatiotemporal evolution of the carbon structure within the pellet depended on the coupling effects of heat and mass transfer with the aromatization reaction [23,55].

#### 3.2.2. Spatiotemporal Evolution of Functional Groups from FTIR Spectroscopy

**Figure 8:**
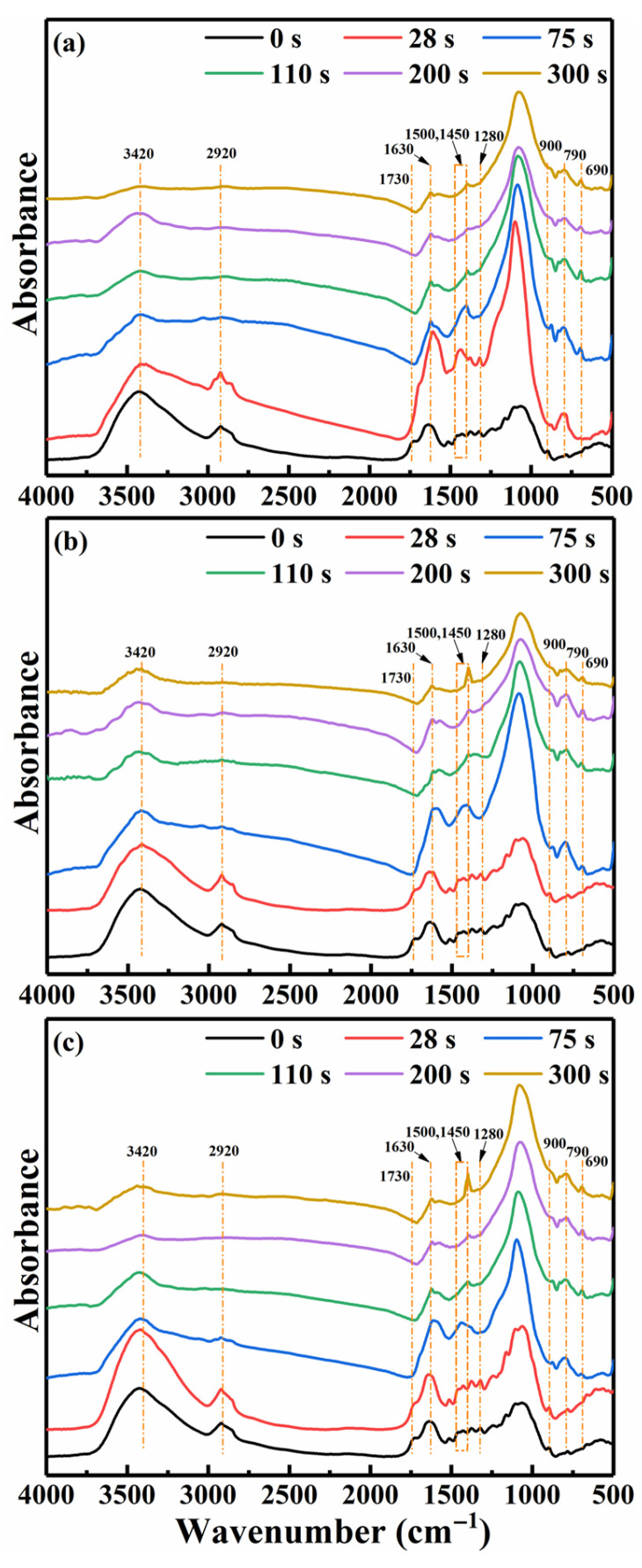
FTIR spectra of raw rice straw and chars at locations (**a**) T, (**b**) TC, and (**c**) C.

Figure 8 shows the FTIR spectra of the raw rice straw and chars collected at locations T, TC, and C versus pyrolysis time. The corresponding FTIR absorption peak assignments are summarized in Table 3. The variations in functional groups (e.g., hydroxyl, carbonyl, alkyl C-H, olefinic C=C, and aromatic C=C) at different stages and locations within the pellet also reflected the spatiotemporal evolution of the chemical structure in the char.

Complete pellet conversion at 700 °C occurred within 75 s, while the center location C required 110 s to attain the target temperature. The peak intensity of the hydroxyl group (3420 cm^−1^) progressively decreased with increasing pyrolysis time. The peaks corresponding to aliphatic symmetric CH_2_ stretching (2920 cm^−1^), C=O stretching (1730 cm^−1^) in esters or carboxylic acids, and C-O deformation (1280 cm^−1^) in phenols were diminished at 75 s. The observed variations in peak intensity were primarily attributed to the elevated pyrolysis temperature, which promoted the cleavage of weaker aliphatic bridging bonds and the generation of molecular fragments, thereby facilitating the release of substantial amounts of hydrogen- and oxygen-containing volatiles [56,57]. Moreover, increased temperature also promoted the demethoxylation, demethylation, and dehydration reactions of lignin in the biomass, thereby reducing the abundance of unstable aliphatic compounds during pyrolysis [58,59]. In response, the intensity of the C=C stretching peak at 1630 cm^−1^ in the olefin structures initially increased during the first 110 s and then gradually decreased from 110 to 300 s. During the later stage of pellet pyrolysis, the decrease in the intensity of the C=C stretching peak at 1630 cm^−1^ was attributed to the formation of small aromatic ring structures through cyclization and aromatization reactions [60,61]. Subsequently, these small aromatic rings underwent condensation reactions at active sites, originating from unsaturated functional groups, to form large aromatic ring structures [23]. Importantly, the aromatic ring skeletal C=C stretching vibration, observed as a band near 1500 cm^−1^, shifted to lower wavenumbers and eventually split into two distinct peaks at approximately 1500 cm^−1^ and 1450 cm^−1^. This splitting was related to the conjugation effect between the benzene ring and adjacent electron-withdrawing or electron-donating groups, such as carbonyl (C=O), vinyl (C=C), and nitrile (C≡N) [62]. Thus, the formation of highly condensed aromatic ring systems facilitated the growth of more stable carbon structures.

The differences in infrared peak intensity at different locations indicated a limited spatial heterogeneity in the evolution of functional groups, likely resulting from the close location distances. The changes in the 1000–1800 cm^−1^ bands occurred first at location T (28 s), with a noticeable time delay observed at locations TC and C (75 s or later). The aliphatic symmetric CH_2_ stretching peak (2920 cm^−1^) in the char from location T disappeared by 75 s, whereas the corresponding peak in the char from location C persisted until 110 s. Notably, a similar phenomenon was also observed for the aromatic ring skeletal C=C stretching vibration at locations T, TC, and C. The intensity of this C=C peak in the char from location T initially increased transiently and then gradually decreased, nearly disappearing in the later stage. Unlike location T in the later stage, the peak intensities in the char from locations TC and C did not disappear. The initial increase was attributed to the rapid heating of the outer surface, whereas the subsequent decrease was primarily associated with volatile release and the aromatization of the deposited volatiles, driven by the coupling effects of heat and mass transfer [23,55]. The variations in functional group peak intensities revealed the temporal and spatial evolution of the chemical structure in the char at locations T, TC, and C within a 10 mm-diameter pellet.

### 3.3. Alkali and Alkaline Earth Metal Contents Within the Pellet

Table 4 shows the contents of alkali and alkaline earth metals at locations T, TC, and C within the pellet during pyrolysis at 700 °C, based on our previous work [38]. It was observed that the sodium content was extremely low, and calcium and magnesium were not easily released. Thus, potassium dominated the catalytic effects of alkali and alkaline earth metals on char conversion. Furthermore, a slower heating rate and greater mass transfer resistance at the inner layer location C limited potassium release at 75 s, resulting in a distinct spatial distribution. As pyrolysis progressed to 300 s, this spatial difference gradually diminished with the homogenization of intra-pellet temperature distribution.Spatially heterogeneous potassium distribution during the initial pyrolysis stage produced distinct catalytic effects on char reactivity at different locations.

### 3.4. Reactivity of Char at Different Stages and Locations Within the Pellet

**Figure 9:**
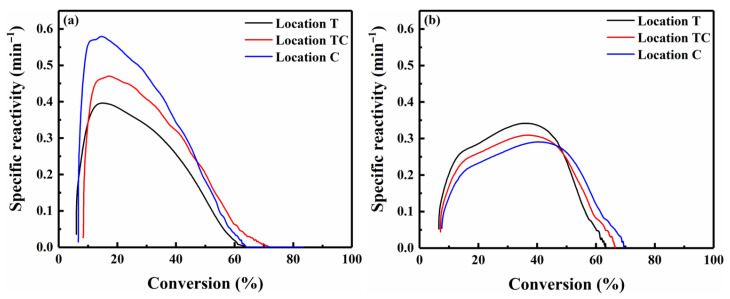
Specific reactivity of char within the pellet for pyrolysis times of (**a**) 75 s and (**b**) 300 s.

Figure 9 presents the specific reactivity curves of chars at distinct stages and locations within the pellet. During char reactivity testing, a thin layer of fine char particles (<50 µm) was used to minimize heat and mass transfer limitations, ensuring that the measured specific reactivity was truly intrinsic reactivity reflecting the char structure. The intrinsic reactivity of the char was measured under a kinetic control regime (reactive gas of 5% O_2_ in N_2_, and reactive temperature of 400 °C) independent of the gas flow rate and particle size [9]. Thus, any differences observed in char reactivity were related to the evolution of the chemical structure in the char [9,10]. Large aromatic systems (≥6 fused rings), as indicated by the D band, demonstrated higher thermal stability and lower reactivity during char conversion, whereas small aromatic structures (3–5 fused rings), represented by the combined (G_r_ + V_l_ + V_r_) band, exhibited higher reactivity [23]. For the char produced after 75 s of pyrolysis, at any given conversion level, the specific reactivity was highest at location C, followed by TC and then T. The increased reactivity was primarily attributed to the higher abundance of aliphatic structures and smaller aromatic ring structures with more reactivity Moreover, higher potassium content at location C further intensified the reactivity, especially the maximum specific reactivity. The specific reactivity of the char at locations T, TC, and C initially increased to a peak of 0.58 during the first ~13% of conversion, followed by a gradual decrease with further conversion. These observed variations indicated that aliphatic structures were preferentially consumed during the initial stage, while small aromatic ring structures were consumed subsequently [20], as illustrated in Figure 8. At 300 s, the increased abundance of large aromatic ring structures (≥6 fused rings) at center location C resulted in a lower char reactivity. These trends are consistent with the variations in the A_D_/A_G_ and A_D_/A_(Gr+Vl+Vr)_ ratios in Figure 7.

## 4. Conclusions

This study experimentally investigates the effects of spatial scale, specifically pellet size and intra-pellet location, on char structural evolution and reactivity. The main conclusions are as follows:

(1) Increasing the pellet size facilitates the growth of polycyclic aromatic structures through cyclization and condensation reactions, as evidenced by variations in the abundance of typical aromatic compounds in bio-oil. Furthermore, it promotes volatile–char interactions, leading to greater deposition of large aromatic structures on the char surface, thereby enhancing char aromatization.
(2) Analogous to the spatial scale effect of pellet size on char structure, the location-dependent coupling of heat/mass transfer limitations and aromatization reactions leads to a significant spatial difference in the relative abundance of large aromatic rings in the char structure: the outer surface (location T) exhibits the highest abundance of large aromatic rings during the devolatilization stage (0–75 s), whereas the center (location C) becomes dominant in the later char aromatization stage (110–300 s). Additionally, variations in infrared peak intensity on aromatic functional groups provide supplementary insights into the condensation evolution of aromatic structures.
(3) During the devolatilization stage at 75 s, the center location exhibits the highest reactivity, attributed to a greater presence of aliphatic groups and small aromatic rings, whereas the outer surface becomes the most reactive at 300 s due to reduced aromatic condensation.

## Figures and Tables

**Table 1:** Properties of the rice straw sample.

Sample	Proximate Analysis (wt.%, db)			Ultimate Analysis (wt.%, db)				
	Volatile	Ash	Fixed Carbon	C	H	N	S	O*
Rice straw	79.92	13.35	6.73	41.42	5.84	1.71	0.27	37.41

O* by difference.

**Table 2:** Yield distribution of products during different-sized single-pellet pyrolysis at 700 °C.

Sample	Product Yield (wt.%, on a Dry Basis)	Pellet Size (mm)		
		8	10	12
Rice straw	Bio-oil	34.21 ± 1.66	33.11 ± 2.47	31.55 ± 2.98
	Moisture	7.59 ± 1.07	7.70 ± 0.89	8.11 ± 0.34
	Gas	27.50 ± 0.46	27.95 ± 0.57	28.17 ± 0.49
	Char	31.28 ± 0.32	31.41 ± 0.12	32.44 ± 0.78
	Total	100.58 ± 3.51	100.17 ± 4.05	100.27 ± 4.59

**Table 3:** Functional groups in chars of rice straw.

Wavenumber (cm^−1^)	Infrared Absorption	Functional Groups and Structures
3420	O-H stretching vibration	H bond
2920	C-H stretching vibration	C-H bond in aliphatic structures
1730	C=O stretching vibration	C=O bond in esters or carboxylic acid
1630	C=C stretching vibration	C=C bond in olefin structures
1500, 1450	C=C stretching vibration	C=C bond in aromatic ring structures
1280	C-O deformation vibration	C-O bond in phenol
900	C-H deformation stretching	C-H bond in aromatic ring structures
790	C-H deformation stretching	C-H bond in aromatic ring structures
690	C-H deformation stretching	C-H bond in aromatic ring structures

**Table 4:** Distribution of alkali and alkaline earth metals at different stages and locations.

Elements	Raw Rice Straw (mg/g)	Pyrolysis Char (mg/g)					
		75 s			300 s		
		T	TC	C	T	TC	C
Na	0.07	0.050	0.058	0.060	0.053	0.050	0.050
K	15.73	11.054	11.585	11.764	10.555	10.598	10.563
Ca	3.55	3.311	3.365	3.477	3.231	3.242	3.239
Mg	1.66	1.459	1.469	1.543	1.461	1.456	1.463

## Data Availability

The original contributions presented in this study are included in the article. Further inquiries can be directed to the corresponding author.
